# Cellular Senescence and Immunosenescence in Melanoma: Insights From the Tumor Microenvironment

**DOI:** 10.1002/cam4.71223

**Published:** 2025-09-09

**Authors:** Lihua Xiong, Jian Cheng

**Affiliations:** ^1^ Department of Dermatology Cheng Du Xinjin District Hospital of Traditional Chinese Medicine Chengdu China; ^2^ Department of Chinese Medicine Sichuan Provincial People's Hospital, School of Medicine, University of Electronic Science and Technology of China Chengdu China

**Keywords:** cellular senescence, immunosenescence, melanoma, senescence‐associated secretory phenotype, tumor microenvironment

## Abstract

**Background:**

Melanoma is one of the most immunogenic malignancies, yet resistance to immune checkpoint inhibitors (ICIs) remains a major obstacle to durable therapeutic success. Emerging evidence indicates that aging‐related processes, including cellular senescence and immunosenescence, can reshape the tumor microenvironment (TME) to favor immune evasion and disease progression. Senescent melanoma and stromal cells secrete a senescence‐associated secretory phenotype (SASP) that alters immune cell recruitment and function, while immunosenescence leads to diminished cytotoxic responses and the accumulation of dysfunctional or suppressive immune subsets.

**Aim:**

This review explores the interplay between cellular senescence and immunosenescence in melanoma, highlighting their contributions to tumor progression and immunotherapy resistance, and discusses potential strategies to therapeutically target senescence‐related pathways.

**Methods:**

A systematic review of studies published between 2000 and 2024 was performed using PubMed, Web of Science, and Scopus. Literature included mechanistic investigations of senescence in melanoma, analyses of immunosenescence in cancer patients, and preclinical or translational studies targeting senescence‐related pathways.

**Results and Conclusions:**

Senescent tumor and stromal cells drive a pro‐inflammatory and immunosuppressive TME through SASP, while aging immune cells exhibit impaired antigen presentation, reduced cytotoxicity, and increased suppressive subsets. These dual processes form a self‐reinforcing cycle of chronic inflammation and immune dysfunction, ultimately undermining the efficacy of ICIs. Targeting senescence, through senolytics, senostatics, or SASP modulators, has shown promise in preclinical models and may restore immune competence in melanoma. However, clinical translation requires further investigation to validate safety and efficacy. Addressing both cellular and immune senescence represents a novel and promising direction to overcome therapeutic resistance and improve melanoma outcomes.

## 1 Introduction

Melanoma is one of the most aggressive forms of skin cancer, characterized by high metastatic potential, pronounced intratumoral heterogeneity, and notable immunogenicity [1, 2]. Epidemiological studies have shown that the incidence of melanoma increases with age, and older patients often exhibit distinct tumor‐immune interactions compared with younger individuals [3, 4]. Despite the clinical success of immune checkpoint inhibitors (ICIs), including anti‐CTLA‐4 and anti‐PD‐1 antibodies, a substantial proportion of patients either fail to respond or relapse after an initial response [5, 6]. Multiple factors have been proposed to contribute to this variability, among which the tumor immune microenvironment (TIME) has emerged as a critical determinant of therapeutic outcomes [7]. In this context, increasing attention is being directed toward aging‐associated cellular processes that shape the melanoma immune landscape and influence both disease progression and immunotherapy responsiveness.

Cellular senescence is a stable form of proliferative arrest that occurs in response to a variety of stressors, including oncogene activation, telomere attrition, genotoxic damage, oxidative stress, and therapeutic interventions [8]. Senescent cells remain metabolically active and display characteristic features such as the expression of p16 (INK4a), p21 (CIP1), senescence‐associated β‐galactosidase (SA‐β‐gal), and nuclear envelope disruption [9, 10]. A hallmark of senescence is the acquisition of a senescence‐associated secretory phenotype (SASP), comprising cytokines, chemokines, growth factors, and proteases [11, 12]. These factors modulate the local microenvironment by regulating immune cell recruitment and function, remodeling the extracellular matrix, and influencing angiogenesis. In melanoma, senescence may arise as a result of oncogenic BRAF or NRAS activation, therapeutic stress, or immune‐mediated signals [13, 14]. Importantly, senescence exerts context‐dependent effects: acute senescence may limit tumor growth via cell cycle arrest and immune clearance, whereas persistent accumulation of senescent cells has been linked to chronic inflammation, immune suppression, and therapeutic resistance [15].

Alongside tumor cell senescence, aging is accompanied by gradual deterioration of immune competence, a process termed immunosenescence. This is characterized by impaired cytotoxic function, skewed cytokine production, reduced antigen presentation, and expansion of dysfunctional or suppressive immune subsets [16]. In melanoma, immunosenescence has been associated with diminished efficacy of ICIs and altered composition of tumor‐infiltrating lymphocytes in elderly patients [17]. Moreover, senescent immune cells can amplify local inflammatory signaling and contribute to the establishment of an immunosuppressive niche. Evidence suggests that cellular senescence and immunosenescence are not independent but instead co‐evolve within the tumor microenvironment (TME), forming a regulatory axis that shapes immune evasion and tumor progression [18]. In this review, we distinguish between senescent tumor cells, which undergo stable cell cycle arrest and SASP production, and senescent immune cells, marked by functional exhaustion and impaired surveillance. Rather than evolving independently, these two compartments form an interactive regulatory axis that shapes immune suppression and tumor progression in aged melanoma.

Over the past decade, mounting evidence has revealed how age‐associated cellular programs impact melanoma pathogenesis and treatment response. Aged melanoma patients frequently exhibit lower tumor mutational burden, impaired antigen presentation, and altered T cell functionality, contributing to reduced immunotherapy efficacy [19, 20]. Meanwhile, senescent cells in the tumor and stromal compartments persistently release SASP factors that reinforce immune evasion and metastatic dissemination [21, 22]. Yet, the convergence of these aging‐related phenomena in shaping the immunological landscape of melanoma remains incompletely defined. In this review, we synthesize emerging insights into the mechanisms and consequences of cellular senescence and immunosenescence in melanoma, with particular emphasis on their intersection within the TME. We first dissect how senescent cells modulate immune surveillance, then examine how immunosenescence reshapes immune functionality in aged melanoma, and finally explore therapeutic strategies aimed at rejuvenating anti‐tumor immunity through modulation of senescence‐related pathways.

## 2 Age‐Associated Features of Melanoma

### 2.1 Epidemiological Trends and Aging‐Associated Melanoma Risk

Melanoma is one of the most immunogenic malignancies, and its incidence has been shown to increase with age [23]. Epidemiological studies have consistently identified age as a significant prognostic factor across various disease stages. A retrospective analysis demonstrated that older patients had significantly poorer melanoma‐specific survival compared with younger individuals, even after the development of distant metastases [24]. Specifically, 2‐year survival rates were 34.8% for patients under 40 years of age, 21.2% for those aged 40–59.9 years, and 10.9% for patients aged 60 years and above [25]. Moreover, advanced age was associated with a higher risk of in‐transit metastases and nodal recurrences, further supporting the impact of aging on disease progression [25]. In addition to its prognostic relevance, aging also influences the biology of melanoma through changes in the TME. These include alterations in immune cell composition and function that may promote tumor development and impair responses to therapy [26]. As melanoma incidence continues to rise in aging populations, understanding how aging‐related processes contribute to disease onset, progression, and therapeutic vulnerability has become increasingly important.

### 2.2 Age‐Related Decline in Anti‐Tumor Immunity

Aging is accompanied by progressive functional impairments of both the innate and adaptive immune systems, collectively referred to as immunosenescence. These alterations are particularly relevant in melanoma, a cancer type highly dependent on immune surveillance and immune‐mediated control. In elderly individuals, T cell populations exhibit phenotypic and functional shifts, including reduced naïve T cell output, accumulation of memory and exhausted T cells, and diminished proliferative and cytotoxic capacity [27]. Aged CD8^+^ T cells show impaired recognition of tumor antigens and reduced interferon‐γ production, limiting their ability to eliminate malignant cells effectively [27]. Beyond T cells, aging also affects dendritic cells (DCs), natural killer (NK) cells, and macrophages. Aged DCs demonstrate reduced capacity to process and present antigens, while NK cells show diminished cytotoxic function, all of which compromise tumor immune surveillance [28]. Furthermore, aging is associated with an increase in suppressive immune subsets, such as regulatory T cells (Tregs) and myeloid‐derived suppressor cells (MDSCs), which contribute to an immunosuppressive TME and hinder effective anti‐tumor responses [29, 30]. These immunological changes have tangible consequences for therapy. Elderly patients with melanoma often exhibit reduced responsiveness to immune checkpoint blockade, potentially due to the altered composition and functionality of their immune repertoire [31]. While ICIs have transformed the treatment landscape, age‐related immune dysfunction may partially account for the variability in clinical outcomes observed across different age groups.

### 2.3 Clinical Implications for Immunotherapy in Elderly Melanoma Patients

The advent of ICIs has transformed the treatment landscape of advanced melanoma [5]. However, patient responses vary widely, and accumulating clinical evidence suggests that aging may influence immunotherapy outcomes [32]. Elderly patients are under‐represented in clinical trials, yet real‐world data have begun to provide insight into the efficacy and tolerability of ICIs in this population.

While some studies have shown comparable overall survival and progression‐free survival between younger and older patients, others report reduced clinical benefit in the elderly, particularly among those with pre‐existing immune dysfunction or comorbidities [25]. These discrepancies may reflect heterogeneity in biological age, immune competence, and tumor features that are not captured by chronological age alone. Importantly, elderly patients often exhibit increased frequencies of immunosuppressive cell populations, altered cytokine profiles, and diminished tumor‐infiltrating lymphocyte (TIL) activity, all of which may compromise anti‐tumor immunity and reduce ICI efficacy [33]. Furthermore, although the safety profile of ICIs in older individuals is generally manageable, age‐related physiological decline may increase the risk of immune‐related adverse events (irAEs) and affect treatment tolerance [34]. The presence of age‐associated comorbidities also complicates clinical decision‐making and underscores the need for more refined biomarkers to predict treatment outcomes in elderly patients. Taken together, these findings highlight the necessity of integrating aging‐related biological factors into the clinical management of melanoma. Personalized strategies that account for immune competence, tumor immunogenicity, and patient frailty may improve treatment outcomes in older individuals.

## 3 Cellular Senescence in Melanoma

### 3.1 Mechanisms Inducing Senescence in Melanoma

**FIGURE 1:**
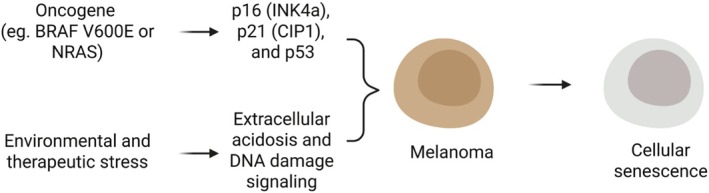
Induction of cellular senescence in melanoma by oncogenic and environmental stressors. Melanoma cells undergo senescence upon exposure to intrinsic oncogenic signals or extrinsic stressors. Activating mutations in oncogenes such as BRAF^V600E^ or NRAS trigger the upregulation of canonical cell cycle inhibitors including p16(INK4a), p21(CIP1), and p53. In parallel, environmental and therapeutic stress—such as extracellular acidosis and genotoxic insults—can also promote senescence through DNA damage signaling.

Aging melanocytes and melanoma cells exhibit divergent senescence trajectories. While normal melanocytes primarily undergo replicative senescence via telomere attrition, melanoma cells acquire oncogene‐induced senescence (OIS) that can be bypassed or reversed under selective pressure (Figure 1). Cellular senescence is a stress‐responsive and durable cell cycle arrest program that can be triggered by a variety of intrinsic and extrinsic stimuli [35]. In melanoma, OIS is one of the earliest discovered mechanisms [36]. Mutations in BRAF V600E or NRAS, common drivers in melanoma, can paradoxically induce senescence in melanocytes during early transformation stages. This form of OIS is characterized by activation of p16 (INK4a), p21 (CIP1), and p53, as well as chromatin remodeling and metabolic rewiring [37, 38]. Although OIS initially restrains malignant progression, tumor cells often acquire secondary genetic or epigenetic alterations that allow them to bypass this barrier. In addition to oncogene activation, melanoma cells can enter senescence in response to environmental and therapeutic stress. Extracellular acidosis, a hallmark of the TME, has been shown to induce a senescence‐like phenotype in human melanoma cells [39]. This is associated with persistent DNA damage signaling, nuclear morphological alterations, and the induction of inflammatory mediators such as CXCL8 and IL‐1α. Similarly, therapy‐induced senescence (TIS) occurs following treatment with BRAF or MEK inhibitors, ionizing radiation, or immunotherapies [40]. Notably, TIS does not always represent a permanent arrest. Senescent melanoma cells can adopt plastic states and potentially re‐enter the cell cycle, a phenomenon that may contribute to disease relapse and therapeutic resistance [41].

### 3.2 Functional Duality of Senescence and SASP‐Related Microenvironmental Remodeling

Senescence exerts context‐dependent effects on tumor progression. On one hand, acute senescence acts as a tumor‐suppressive mechanism by halting cell proliferation and activating immune‐mediated clearance [42]. On the other hand, chronic accumulation of senescent cells in the TME may promote inflammation, matrix remodeling, and immunosuppression, collectively facilitating tumor progression [43]. A key feature mediating these divergent outcomes is the SASP, a complex mixture of cytokines, chemokines, growth factors, and proteases. In melanoma, SASP components such as IL‐6, IL‐8, and MMPs have been implicated in promoting angiogenesis, epithelial‐mesenchymal transition, and recruitment of MDSCs [44, 45]. The composition and intensity of the SASP can vary depending on the senescence inducer, cell type, and duration of the response. Moreover, SASP secretion is dynamically regulated by the NF‐κB and C/EBPβ pathways and can be modulated by the TME [46, 47]. Persistent SASP expression may establish a chronic inflammatory milieu that supports tumor cell survival and reshapes immune cell infiltration.

### 3.3 Therapy Resistance and Immune Consequences of Persistent Senescence

Senescent melanoma cells can influence treatment responses through multiple mechanisms. TIS contributes to a temporary reduction in tumor burden; however, the long‐term presence of senescent cells has been associated with therapy resistance [48]. SASP factors can stimulate tumor cell dedifferentiation, promote survival pathways, and alter the phenotype of neighboring cells, creating a microenvironment conducive to tumor recurrence. From an immunological perspective, senescent cells can either stimulate or suppress anti‐tumor immunity. While acute senescence may enhance immunogenicity through the release of damage‐associated molecular patterns (DAMPs) and chemokines that recruit cytotoxic lymphocytes, persistent SASP expression may shift this balance toward immunosuppression [49]. SASP‐driven attraction of MDSCs and Tregs, along with inhibition of DCs function, has been observed in melanoma models [50]. Furthermore, the pro‐inflammatory but immunosuppressive environment generated by senescent cells may impair the efficacy of ICIs [51]. These observations suggest that senescent cells not only influence tumor biology but also represent a barrier to durable therapeutic responses. Strategies aimed at selectively eliminating senescent cells or modulating the SASP, known as senolytic or senomorphic approaches, have shown promise in preclinical melanoma models [52]. Nevertheless, understanding how senescence contributes to the immunological landscape of melanoma may provide new opportunities to improve immunotherapy outcomes.

## 4 Aging Immune Landscape in Melanoma

**FIGURE 2:**
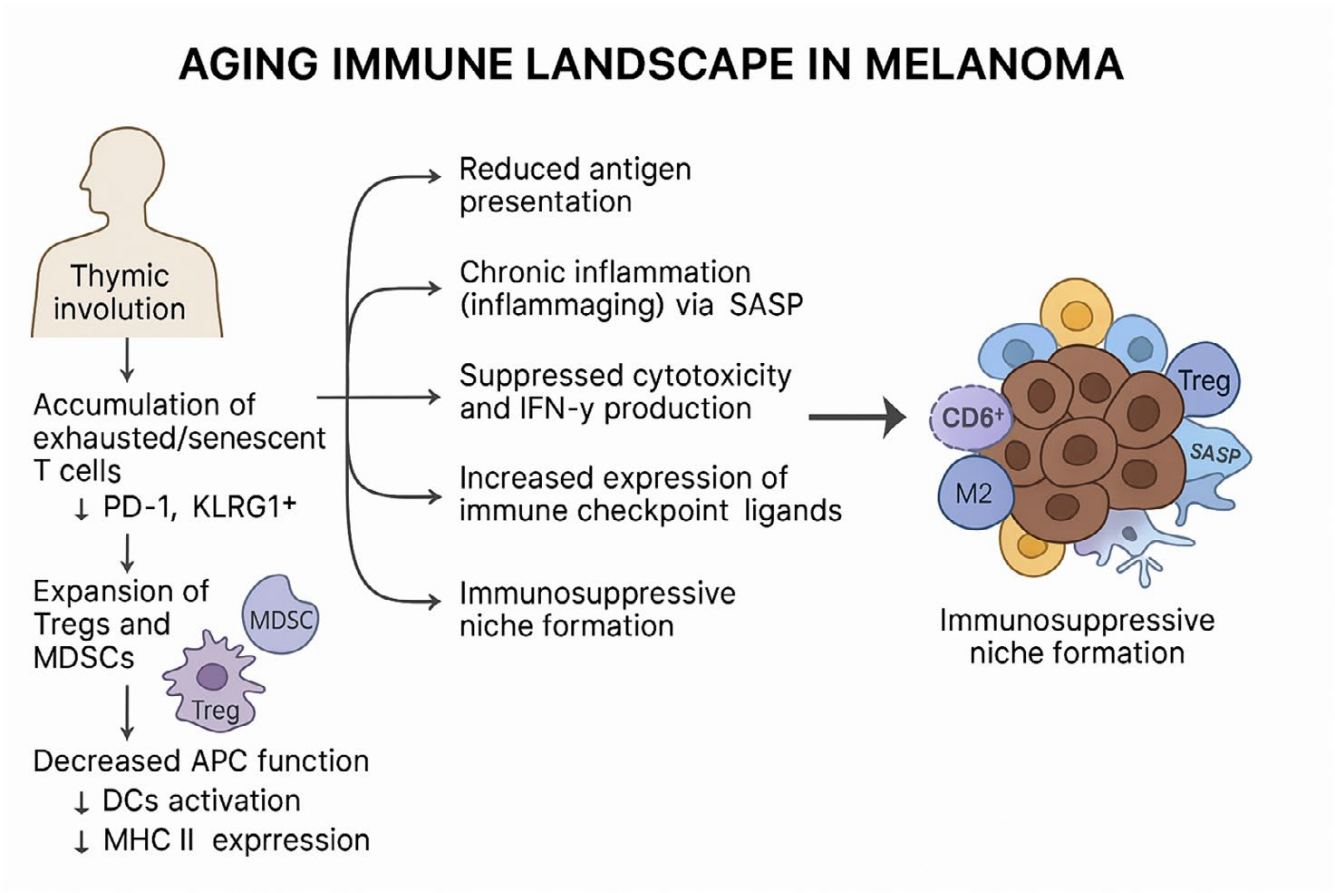
Remodeling of the aged immune landscape in melanoma. Aging‐associated immune alterations contribute to the formation of an immunosuppressive tumor microenvironment in melanoma. These changes include impaired dendritic cell activation and antigen presentation, accumulation of senescent and exhausted CD8^+^ T cells, reduced natural killer (NK) cell cytotoxicity, and expansion of suppressive myeloid populations such as M2‐like tumor‐associated macrophages (TAMs) and myeloid‐derived suppressor cells (MDSCs).

Age‐related decline in immune competence is a well‐established phenomenon, commonly referred to as immunosenescence. This multifaceted process is characterized by impaired lymphocyte function, altered innate immune responses, and chronic low‐grade inflammation [16, 53]. In melanoma, the consequences of immunosenescence are especially pronounced, given the disease's immunogenic nature and its dependency on immune surveillance (Figure 2). In this section, we discuss the key immune cell populations affected by aging, their roles in shaping the TIME, and the therapeutic implications in elderly melanoma patients.

### 4.1 Remodeling of Myeloid Compartment in Aged Melanoma

Aging profoundly alters the composition and function of myeloid cells in the TME, which includes DCs, tumor‐associated macrophages (TAMs), and MDSCs. These changes collectively reshape the immune landscape of melanoma in elderly individuals and contribute to immune evasion and resistance to immunotherapy.

DCs play a central role in initiating anti‐tumor responses through antigen presentation and T cell priming [54]. However, aging is associated with both quantitative and qualitative defects in DC populations [55]. Age‐related reductions in the number of Langerhans cells and dermal DCs have been reported in human skin, which may impair local immunosurveillance [28]. In addition, DCs from aged hosts show diminished expression of co‐stimulatory molecules (such as CD80 and CD86), reduced migratory capacity, and impaired production of IL‐12, all of which compromise their ability to prime naïve T cells [56]. These defects have been shown to limit the generation of cytotoxic CD8^+^ T cell responses against melanoma antigens, thereby facilitating tumor progression in aged hosts. In preclinical models, DC‐based vaccines exhibited lower efficacy in aged mice compared with young counterparts, highlighting the impact of senescent DC networks on therapeutic responsiveness [57].

In aged melanoma microenvironments, there is an increased accumulation of immunosuppressive myeloid subsets, notably TAMs with an M2‐like phenotype and MDSCs [58]. Aging skews monocyte differentiation toward anti‐inflammatory macrophages expressing high levels of Arg1, IL‐10, and TGF‐β. These M2‐like TAMs actively suppress T cell effector functions, promote tissue remodeling, and facilitate angiogenesis. Concurrently, MDSCs are expanded in the circulation and within tumors of elderly patients. These cells inhibit T cell proliferation via nitric oxide production and arginine depletion and impair NK cell activation [30]. Multiple studies have demonstrated that age‐related expansion of MDSCs correlates with resistance to immune checkpoint blockade, particularly anti‐PD‐1 therapy [59]. Importantly, these suppressive myeloid populations are not static but dynamically shaped by the TME and systemic inflammatory cues. Taken together, these findings underscore that myeloid cells are both targets and mediators of senescence‐associated immune dysfunction in melanoma. A deeper understanding of their age‐specific regulation will be essential for designing more effective immunotherapeutic approaches tailored to elderly patients.

### 4.2 Exhaustion and Dysfunction of T Lymphocytes

NK cells and CD8^+^ T cells represent two pivotal cytotoxic arms of anti‐tumor immunity. In melanoma, both populations contribute to direct tumor cell killing, cytokine production, and the orchestration of broader immune responses [60]. However, aging profoundly alters the phenotype and functionality of these cell types, compromising their ability to control tumor progression and respond to immunotherapy.

In elderly individuals, NK cells display reduced cytotoxic potential, impaired cytokine secretion, and diminished proliferative capacity [61]. Phenotypic analyses have revealed a skewing toward the CD56 dim CD16^+^ terminally differentiated subset with lower expression of activating receptors such as NKG2D and NKp30, accompanied by increased expression of inhibitory receptors including KLRG1 and NKG2A [45]. Functionally, these alterations lead to reduced granzyme B release and defective target recognition in melanoma‐bearing hosts. Tumor‐derived factors such as TGF‐β and IL‐10 exacerbate NK dysfunction by dampening receptor expression and altering metabolic fitness. Notably, melanoma cells within an aged microenvironment were found to be less susceptible to NK‐mediated lysis, suggesting a tumor‐intrinsic adaptation to immune aging [45]. In parallel, CD8^+^ T cells undergo extensive remodeling during aging. Senescent CD8^+^ T cells accumulate in peripheral tissues and tumors, characterized by the loss of CD28 expression, upregulation of CD57 and KLRG1, and impaired TCR signaling [62]. While senescent CD8^+^ T cells may share features with exhausted cells, including impaired cytotoxicity and checkpoint expression, they are typically non‐proliferative and metabolically distinct, supporting a classification of true senescence rather than reversible exhaustion. These cells exhibit reduced proliferative capacity and cytokine production, limiting their ability to sustain effective anti‐tumor responses. In melanoma, immune aging is associated with decreased clonal diversity of tumor‐infiltrating CD8^+^ T cells and increased expression of exhaustion markers such as PD‐1, LAG‐3, and TIM‐3 [26]. Although checkpoint blockade aims to reinvigorate exhausted T cells, aged CD8^+^ T cells often fail to mount robust effector responses due to defective co‐stimulation and diminished metabolic plasticity. Importantly, recent findings have challenged the notion that senescent T cells are functionally inert. In certain contexts, senescent‐like CD8^+^ T cells acquire innate‐like features reminiscent of NK cells, including expression of NKG2D and cytotoxic granules, which may confer residual or alternative effector functions. These phenotypic alterations suggest that senescent CD8^+^ T cells may exhibit both impaired effector function and a gain of innate‐like, pro‐inflammatory properties. While such duality may seem contradictory, it reflects a context‐dependent spectrum of activity: in some settings, these cells may transiently retain cytotoxicity; in others, their pro‐inflammatory but dysfunctional state may exacerbate immune suppression. Future studies are warranted to dissect their net contribution in aged melanoma TME. However, whether these adaptations support or hinder anti‐melanoma immunity in aged hosts remains incompletely defined and likely varies across tumor stages and treatment contexts.

### 4.3 Therapeutic Resistance in Elderly Patients

Despite the transformative impact of ICIs in melanoma treatment, their efficacy remains variable across age groups. Older patients with melanoma frequently exhibit diminished responses to immunotherapy, a phenomenon increasingly attributed to age‐associated alterations in immune architecture, stromal composition, and tumor‐host interactions.

Immunosenescence alters both the quantity and quality of T cells, reducing the pool of naïve T cells while expanding terminally differentiated effector memory cells with reduced proliferative potential [63]. In melanoma, aged patients often display impaired expansion of tumor‐specific CD8^+^ T cells following ICI therapy, accompanied by upregulation of exhaustion markers such as PD‐1, LAG‐3, and TIM‐3 [64]. These senescent‐like T cells exhibit reduced cytokine production and cytotoxic capacity [64]. Moreover, recent evidence suggests that the clonal diversity of T cells in elderly patients is more restricted, limiting the breadth of tumor‐reactive responses [65]. For instance, in anti‐PD‐1‐treated melanoma cohorts, diminished peripheral T cell reinvigoration in aged individuals was predictive of inferior clinical outcomes [66]. As detailed earlier, aging promotes the accumulation of immunosuppressive TAMs and MDSCs within the TME. These cells not only dampen T cell responses through metabolic and cytokine‐mediated mechanisms but also interfere with effective antigen presentation. DCs from aged hosts exhibit defective expression of MHC class I and co‐stimulatory molecules, undermining the priming of anti‐tumor T cells [67]. The convergence of poor antigen presentation and enhanced immunosuppression skews the balance toward immune escape. Preclinical studies have shown that blocking IL‐10 or CSF1R in aged melanoma‐bearing mice can partially restore responsiveness to checkpoint blockade, underscoring the central role of myeloid dysfunction in therapeutic resistance [68].

Inflammaging, driven by senescent immune and stromal cells, contributes to a desensitized immune environment that favors tumor persistence. Elevated systemic levels of IL‐6, TNF‐α, and CRP are frequently observed in older patients and correlate with poorer responses to ICIs [69]. In addition, aging‐associated changes in the extracellular matrix and fibroblast phenotype alter immune cell trafficking and may impede effective T cell infiltration into tumor cores [70]. Melanoma lesions in elderly individuals often harbor fibroblast‐enriched desmoplastic zones and exhibit increased expression of chemokines such as CCL2 and CXCL12, which recruit suppressive macrophages and exclude effector lymphocytes [71].

## 5 Crosstalk Between Tumor and Immune Senescence

### 5.1 SASP‐Mediated Modulation of Immune Cell Function in Aged Melanoma

Senescent cells, although irreversibly growth arrested, remain metabolically active and secrete a diverse array of pro‐inflammatory and tissue‐remodeling factors collectively termed the SASP. In melanoma, SASP has emerged as a critical mediator of tumor‐immune microenvironment remodeling, particularly under conditions of aging or chronic stress [72, 73]. These secreted molecules, including interleukins (e.g., IL‐6, IL‐8), chemokines (e.g., CCL2, CXCL10), matrix metalloproteinases (MMPs), and growth factors (e.g., VEGF), exert both local and systemic effects on immune surveillance, contributing to immune dysfunction in aged melanoma microenvironments [74]. In aged melanoma tissues, SASP factors have been shown to impair the recruitment and cytotoxic function of CD8^+^ T cells, either through direct paracrine suppression or by promoting the expansion of suppressive myeloid cells such as MDSCs and M2‐like TAMs. For example, elevated IL‐6 and CCL2 in BRAF‐inhibitor‐induced TIS models of melanoma correlated with increased infiltration of immunosuppressive macrophages, which dampened anti‐tumor T cell activity [75]. In addition, persistent SASP production can drive the expression of checkpoint ligands such as PD‐L1 on myeloid cells, reinforcing immune evasion and contributing to resistance against ICIs [76]. Beyond T cells, SASP factors can also reprogram DCs, impairing their antigen presentation capacity and promoting tolerogenic phenotypes. For instance, SASP‐induced secretion of prostaglandin E2 (PGE2) and IL‐10 was reported to downregulate MHC‐II and costimulatory molecules on DCs, thereby hampering the priming of naïve T cells and limiting the efficacy of tumor‐specific immune responses [77]. Importantly, the impact of SASP on immune modulation appears to be temporally and contextually regulated. Acute SASP may initially aid in immune recruitment and senescent cell clearance, whereas chronic or unresolved SASP sustains a low‐grade inflammatory environment (inflammaging) that reinforces immune exhaustion [77]. This dichotomy is particularly relevant in elderly melanoma patients, where the immune system is already compromised by immunosenescence, amplifying the suppressive effects of SASP through converging molecular pathways [72]. These findings underscore the central role of SASP in linking tumor cell senescence to immune dysfunction in melanoma. By modulating multiple immune cell types and shaping the inflammatory tone of the TME, SASP not only fosters immune evasion but also establishes a feedback loop that entrenches immunosenescence, presenting significant obstacles to effective immunotherapy in aged individuals.

### 5.2 Senescence Immune Cells as Sources of Chronic Inflammation and Immune Escape

With advancing age, not only do tumor cells undergo senescence, but components of the immune system also progressively acquire senescent traits. Senescent immune cells, including CD8^+^ T cells, NK cells, and myeloid populations, accumulate in aged individuals and contribute to the phenomenon of immunosenescence. These cells often exhibit diminished effector functions, altered surface phenotypes, and, notably, a distinct secretory profile akin to the SASP. This immune‐derived SASP constitutes a potent source of chronic, low‐grade inflammation that both reshapes the melanoma TME and promotes immune evasion [78].

Senescent CD8^+^ T cells in elderly individuals often display a terminally differentiated effector memory RA^+^ (TEMRA) phenotype, characterized by expression of CD57 and KLRG1, loss of CD28, and reduced proliferative capacity [79]. While these cells may transiently retain cytotoxic granules, their sustained functionality is impaired. More critically, these T cells secrete elevated levels of pro‐inflammatory cytokines such as IFN‐γ, TNF‐α, and IL‐6, which can support tumor‐promoting inflammation and alter the balance of immune‐stimulatory versus suppressive cues in the melanoma microenvironment [80]. In NK cells, aging is associated with reduced expression of activating receptors (e.g., NKG2D), impaired degranulation, and lowered IFN‐γ production, but simultaneously increased release of pro‐inflammatory mediators [81]. These dysfunctional NK cells fail to effectively eliminate senescent tumor cells or antigen‐low melanoma variants, thereby allowing tumor progression in an immunologically permissive niche.

Beyond lymphoid cells, senescent myeloid cells also exhibit functional skewing toward immunosuppressive phenotypes. For example, senescent macrophages in aged melanoma‐bearing mice upregulate arginase‐1 and IL‐10, suppressing T cell activity and promoting Treg recruitment [44]. These cells also secrete chronic SASP‐like cytokines (e.g., IL‐1β, IL‐8), sustaining a state of inflammaging that promotes tumor progression and therapy resistance [44]. Mechanistically, chronic inflammation driven by senescent immune cells promotes immune checkpoint upregulation on both immune and tumor cells. Elevated IL‐6 and TNF‐α levels have been shown to increase PD‐L1 expression on melanoma cells and MDSCs, thereby reinforcing an immunosuppressive tumor milieu [82]. Moreover, the accumulation of reactive oxygen species (ROS) and persistent activation of NF‐κB signaling in senescent immune subsets further amplifies inflammatory signaling loops, sustaining immunosuppressive cytokine production even in the absence of external stimuli [83]. Thus, senescent immune cells represent not only a failure of immune surveillance but an active source of tumor‐promoting inflammation and immunosuppression.

### 5.3 Metabolic and Epigenetic Interface of Dual Senescence Pathways

The interplay between cellular senescence and immunosenescence in melanoma is tightly orchestrated by metabolic and epigenetic regulators. These regulatory layers not only determine the onset and persistence of senescence but also shape the immune landscape of aging tumors by modulating immune cell functionality and stromal interactions [16]. Metabolically, senescent melanoma cells and immune cells undergo profound reprogramming. Mitochondrial dysfunction, enhanced glycolytic flux, and altered NAD^+^ metabolism are prominent hallmarks of senescent states in both compartments [84, 85]. Accumulation of dysfunctional mitochondria in senescent tumor cells leads to excessive ROS production, a sustained DNA damage response (DDR), and persistent SASP expression, thereby reinforcing chronic inflammation and immune suppression [86]. Similarly, in senescent T cells and NK cells, mitochondrial decay and NAD^+^ depletion impair effector functions and promote an exhausted‐like phenotype [87, 88]. This metabolic convergence impairs the cytotoxic activity of immune effectors while favoring the survival of immunosuppressive subsets such as MDSCs and Tregs within the aged melanoma microenvironment.

Beyond metabolic circuits, epigenetic alterations serve as a second regulatory axis that stabilizes senescence phenotypes. In melanoma, senescent cells accumulate heterochromatin foci (SAHF), marked by histone H3K9 trimethylation and decreased acetylation, contributing to long‐term silencing of cell cycle genes [89]. Importantly, DNA methylation changes in promoter regions of immune‐regulatory genes also modulate SASP output and antigen presentation capacity, affecting how senescent cells are surveilled by the immune system [90]. In parallel, senescent immune cells acquire specific epigenetic signatures, such as loss of H3K27me3 at exhaustion‐related loci or gain of H3K9me3 at inflammatory gene clusters, which fix their dysfunctional state and restrict transcriptional plasticity [91]. These metabolic and epigenetic programs are not isolated but highly interconnected. For example, metabolic shifts such as citrate accumulation or itaconate production can directly influence histone acetylation and DNA methylation by modulating the availability of cofactors (e.g., acetyl‐CoA, α‐ketoglutarate, S‐adenosylmethionine) for chromatin‐modifying enzymes [92]. In melanoma, nutrient‐depleted niches further reinforce these senescence‐associated epigenetic states by altering one‐carbon metabolism and suppressing chromatin remodeling complexes involved in immune gene activation [93]. For example, glucose deprivation in aged TME has been shown to increase histone deacetylation in T cells, reinforcing their epigenetic exhaustion phenotype [94]. Thus, metabolic‐epigenetic feedback loops sustain a senescent TME that favors tumor persistence and immune evasion. Targeting this dual regulatory network offers a promising avenue for rejuvenating anti‐tumor immunity. Inhibitors of epigenetic writers such as EZH2, or metabolic interventions aimed at restoring mitochondrial function or NAD^+^ pools, have been shown to partially reverse senescent traits and enhance immune responsiveness in preclinical melanoma models [95]. However, given the context‐dependent nature of these interventions, especially the risks of SASP reactivation and senescence escape, a more nuanced approach is required. Importantly, both tumor and immune cells undergo coordinated metabolic and epigenetic remodeling during aging. For instance, NAD^+^ depletion not only hampers DNA repair in melanoma cells but also restricts T cell effector functions, reinforcing immune escape. Likewise, epigenetic silencing of IFN signaling pathways has been observed in both exhausted T cells and senescent melanoma cells, revealing convergent escape strategies [96].

## 6 Therapeutic Strategies to Counteract Senescence‐Driven Immune Dysfunction in Melanoma

A variety of therapeutic strategies have been developed to selectively eliminate senescent cells or mitigate their detrimental effects within the TME. These include small‐molecule senolytics targeting anti‐apoptotic pathways, immune‐based approaches enhancing senescent cell clearance, and combination regimens designed to exploit senescence induction followed by removal (Table 1).

**TABLE 1:** Therapeutic strategies targeting senescence‐driven immune dysfunction.

Strategy	Mechanism	Evidence in melanoma	References
Senolytics	Selective elimination of senescent cells by targeting anti‐apoptotic pathways (e.g., BCL‐2 family inhibition)	Navitoclax and fisetin enhance tumor regression by eliminating therapy‐induced senescent cells and improving CD8^+^ T cell infiltration	[97]
Immunotherapeutic clearance of senescent cells	Engineering immune cells (e.g., CAR‐T cells) to recognize and eliminate senescent cells via surface markers like uPAR	uPAR‐targeted CAR‐T cells showed potent senolytic activity in vivo; proof‐of‐concept for melanoma TME	[98]
Epigenetic & metabolic reprogramming	Restoration of transcriptional and metabolic function in aged immune cells (e.g., HDAC or mTOR inhibition)	HDAC inhibitors restored effector cytokines in aged CD8^+^ T cells; metabolic enhancers like NAD^+^ precursors are under study	[99]
Cellular therapies	Adoptive transfer of rejuvenated T cells (e.g., TILs, CAR‐T), supporting persistence and function	Stem‐like TILs from aged melanoma patients retained effector potential post‐expansion	[100]
Senomorphic agents	Suppressing SASP without killing senescent cells to mitigate chronic inflammation	JAK inhibitors and rapamycin reduced SASP and immune suppression in senescent‐rich melanoma models	[101]
Senotherapy–Immunotherapy combinations	Combining senescence clearance or suppression with immune checkpoint blockade to overcome dual immune dysfunction	Navitoclax synergized with anti‐PD‐1; HSP90 inhibitors showed dual senolytic and immunomodulatory roles	[102]

### 6.1 Cleansing the Senescent Tumor Compartment

Senescent cells accumulate with age and in response to therapy, contributing to a dysfunctional TME characterized by persistent inflammation, stromal remodeling, and immune suppression. In melanoma, the presence of senescent tumor or stromal cells may promote immune evasion and reduce therapeutic efficacy. Targeting these cells has thus emerged as a promising strategy to rejuvenate the TME and improve treatment outcomes, especially in elderly patients.

Senolytic drugs selectively eliminate senescent cells by targeting pro‐survival pathways upregulated in senescence. Navitoclax (ABT‐263), an inhibitor of BCL‐2/BCL‐xL, has shown efficacy in clearing therapy‐induced senescent cells in various cancers, including melanoma [103]. However, its clinical application is limited by dose‐limiting thrombocytopenia. Natural compounds such as quercetin and fisetin have demonstrated senolytic activity in preclinical models and are being explored as adjuvant therapies [104]. In melanoma, fisetin has shown the ability to eliminate TIS cells and partially restore T cell infiltration in the TME [105].

Senescent cells can express surface markers such as urokinase‐type plasminogen activator receptor (uPAR), DPP4, or MICA/B, which are recognized by components of the immune system. Strategies to potentiate immune‐mediated clearance of senescent cells include engineering chimeric antigen receptor T (CAR‐T) cells to target senescence‐associated antigens. One recent study engineered CAR‐T cells specific to the uPAR, showing potent senolytic activity in vivo and restoration of tissue homeostasis [106]. Although not melanoma‐specific, this proof‐of‐concept provides a rationale for the development of immunotherapeutic approaches to eliminate senescent cells within the melanoma TME. Several melanoma therapies, including BRAF/MEK inhibitors and radiation, induce a senescence‐like state in both tumor and stromal cells. Sequential combination of senescence‐inducing agents with senolytics may enhance therapeutic efficacy by preventing the protumorigenic effects of the SASP. For example, after senescence induction by palbociclib or vemurafenib, subsequent administration of navitoclax or fisetin led to significant tumor regression in preclinical models [107]. This “one‐two punch” strategy is gaining traction as a means to leverage the cytostatic benefit of senescence without its long‐term drawbacks. Overall, targeting senescent cells holds promise to rejuvenate the melanoma microenvironment, particularly in aged individuals where senescence burden is high. Future directions include identifying melanoma‐specific senescence markers, optimizing combination regimens, and mitigating toxicity associated with senolytic agents.

### 6.2 Reprogramming Immune Cells to Overcome Aging‐Induced Dysfunction

Immunosenescence undermines the anti‐tumor immune response by impairing effector functions, antigen recognition, and cytokine secretion. In melanoma, where ICIs are a cornerstone of therapy, age‐associated immune dysfunction presents a significant barrier to therapeutic success. Emerging evidence suggests that functional restoration or reprogramming of senescent or exhausted immune subsets may reinvigorate anti‐tumor immunity and enhance therapeutic responsiveness in aged patients.

Several strategies are under investigation to rejuvenate dysfunctional immune cells. One approach involves the blockade of inhibitory pathways that are preferentially upregulated in senescent or exhausted T cells. The co‐inhibitory receptors PD‐1, TIM‐3, and LAG‐3 are frequently co‐expressed on senescent CD8^+^ T cells in the melanoma microenvironment of aged individuals [108]. Preclinical studies have demonstrated that combined blockade of these checkpoints can restore cytokine secretion and cytotoxic function of senescent‐like T cells, even in the absence of full clonal expansion [108]. Recent clinical trials also indicate that aged melanoma patients may derive similar or even superior benefits from ICI combinations compared to younger individuals, likely reflecting compensatory immune remodeling under therapeutic pressure [109].

Epigenetic and metabolic interventions provide another axis for immune rejuvenation. Aging is associated with the loss of chromatin plasticity and impaired transcriptional responsiveness in T cells and NK cells. Agents such as histone deacetylase (HDAC) inhibitors or BET bromodomain inhibitors have shown potential to reprogram senescent immune cells by restoring access to effector gene loci [110]. For example, HDAC inhibition in aged CD8^+^ T cells restored IFN‐γ and granzyme B expression, resulting in enhanced melanoma control in mouse models [111]. Additionally, metabolic rewiring of immune cells to promote mitochondrial fitness and glycolytic flexibility can rejuvenate T cell responses [112]. mTOR inhibitors, AMPK activators, and NAD^+^ precursors are currently under exploration in this context.

Cellular therapies offer a promising platform for the rejuvenation of immune function in aged hosts. Adoptive transfer of ex vivo‐expanded tumor‐infiltrating lymphocytes (TILs) or genetically engineered T cells, including CAR‐T and TCR‐T cells, allows for the selection and expansion of functionally competent clones, circumventing endogenous immune senescence. Notably, a subset of TILs derived from elderly melanoma patients retained stem‐like features and potent effector capacity upon in vitro expansion, suggesting that age‐related immune decline is not irreversible [113]. Strategies to promote the survival and persistence of these reinvigorated T cells, including IL‐15 supplementation or modulation of the TGF‐β axis, are being actively pursued. Finally, tissue‐resident immune populations in aged melanoma, such as skin‐resident DCs and macrophages, may be reconditioned to support anti‐tumor immunity. Pharmacologic agents targeting prostaglandin E2 (PGE2) signaling or CSF1R pathways have been shown to reprogram the suppressive myeloid milieu in aged mice, enhancing responsiveness to immunotherapy [114].

### 6.3 Combining Senotherapies and Immunotherapies

Cellular senescence and immunosenescence converge to shape a tumor‐promoting environment that hampers the efficacy of immunotherapies in melanoma [68]. Senescent tumor cells not only resist apoptosis and cytotoxic attack but also release a range of pro‐inflammatory and immunosuppressive factors through the SASP [115]. Concurrently, aged immune cells exhibit functional decline, reducing their ability to mount efficient anti‐tumor responses. These dual barriers necessitate a therapeutic strategy that targets both tumor‐intrinsic senescence and age‐impaired immune surveillance. Combining senolytic agents with immunotherapy has emerged as a compelling approach to address this challenge. Senolytics, such as BCL‐2 family inhibitors (e.g., navitoclax) and FOXO4‐DRI peptides, selectively induce apoptosis in senescent cells by disabling their anti‐apoptotic machinery [116, 117]. In preclinical melanoma models, navitoclax administration resulted in the clearance of TIS tumor cells, dampened SASP production, and enhanced infiltration of functional CD8^+^ T cells, ultimately improving response to anti‐PD‐1 therapy [116]. Similar results were observed with HSP90 inhibitors, which have demonstrated dual senolytic and immunomodulatory properties [118].

Another category of interventions, senomorphics, seeks to suppress the deleterious effects of SASP without eliminating senescent cells [119]. Agents such as rapamycin, metformin, or JAK inhibitors have been shown to blunt SASP production in tumor or stromal compartments, reducing chronic inflammation and myeloid cell recruitment [120, 121]. These modifications can attenuate immune suppression and recondition the TME for improved immune effector function. Of note, JAK1/2 inhibition synergized with ICIs in preclinical models of melanoma characterized by a senescence‐rich microenvironment, suggesting translational potential in elderly patients [122]. However, combining senotherapies with immunotherapy raises concerns. The off‐target effects of senolytics may harm normal senescent cells that contribute to tissue repair or tumor suppression [123]. Moreover, premature clearance of senescent immune cells may impair the delicate balance between tumor clearance and tissue homeostasis, particularly in aged individuals with reduced regenerative capacity [124]. The timing, dosage, and sequence of combined therapy regimens remain poorly defined, and biomarkers for selecting patients most likely to benefit are still under investigation.

Further complicating the landscape is the dual role of senescence in melanoma. While senescence induction has been proposed as a tumor‐suppressive mechanism, particularly following MAPK pathway inhibition, the persistence of senescent melanoma cells may ultimately foster immune evasion and recurrence [115]. Therefore, therapeutic strategies must account for the temporal dynamics of senescence, with careful distinction between transient beneficial senescence and chronic maladaptive senescence. This is especially relevant in elderly melanoma patients, where systemic immunosenescence may limit immune clearance of senescent tumor cells, necessitating pharmacologic intervention. Moving forward, rational combination regimens should integrate senescence‐targeting agents with immunotherapies based on mechanistic insights and patient‐specific aging biomarkers. Precision immunogerontology, an emerging field that integrates age‐related immune remodeling into personalized immunotherapy design, holds promise for optimizing such interventions in elderly melanoma populations. Well‐designed clinical trials with aging‐specific stratification, combined with deep immunoprofiling, will be critical to unlock the full potential of senotherapy–immunotherapy combinations.

## 7 Conclusion

The intertwined processes of cellular senescence and immunosenescence are increasingly recognized as pivotal modulators of melanoma progression and therapeutic response, particularly in the aging population. Senescent tumor and stromal cells reshape the TME through SASP‐mediated signaling, fostering chronic inflammation, stromal remodeling, and immune suppression [125, 126]. Concurrently, age‐related functional decline in DCs, cytotoxic lymphocytes, and myeloid cells compromises antitumor immunity and reduces the efficacy of ICIs. Our review highlights that senescence is not merely a passive consequence of aging but an active driver of immune evasion and resistance in melanoma. This dual role of senescence—as both a tumor‐suppressive mechanism and a promoter of immune dysfunction—underscores the need for therapeutic nuance. The challenge lies in distinguishing beneficial transient senescence from chronic, deleterious forms and designing interventions that restore immune competence without impairing tissue integrity.

Emerging therapeutic strategies targeting senescent cells, modulating SASP components, or reversing immunosenescence offer exciting opportunities to improve outcomes in elderly melanoma patients [127]. However, the implementation of such approaches requires careful consideration of aging‐associated immune heterogeneity, treatment timing, and combinatorial effects. Integration of multi‐omics profiling, aging biomarkers, and patient‐specific immune signatures will be critical to develop rational, age‐tailored immunotherapeutic regimens. As the global population ages, understanding how senescence‐related processes influence tumors is central to the future of cancer therapy. Continued investigation into the molecular and cellular crosstalk between tumor aging and immune decline will be instrumental in realizing precision immunotherapy for older adults with melanoma.

## Author Contributions

L.X. wrote the main manuscript text and prepared Figure 1; J.C. conceived and critically discussed the clinical unmet problems, underlying mechanisms, and wrote the manuscript. All authors have read and approved the final version of the manuscript. All authors read and approved the final manuscript.

## Conflicts of Interest

The authors declare no conflicts of interest.

## Data Availability

The authors have nothing to report.
